# The Association between Diet Quality Scores with Sleep Quality among Employees: A Cross-Sectional Study

**DOI:** 10.4314/ejhs.v32i1.16

**Published:** 2022-01

**Authors:** Hossein Bavi Behbahani, Fatemeh Borazjani, Leila Sheikhi, Rezvan Amiri, Kambiz Ahmadi Angali, Sudabeh Basak Nejad, Mahsa Samadani

**Affiliations:** 1 Student Research Committee, Ahvaz Jundishapur University of Medical Sciences, Ahvaz, Iran; 2 Department of Nutrition, Faculty of Allied Sciences, Ahvaz Jundishapur University of Medical Sciences, Ahvaz, Iran; 3 Nutrition and Metabolic Diseases Research Center and clinical sciences research institute, Ahvaz Jundishapur University of Medical Sciences, Ahvaz, Iran; 4 Nutrition and Metabolic Diseases Research Center and clinical sciences research institute, Ahvaz Jundishapur University of Medical Sciences, Ahvaz, Iran; 5 Department of Biostatistics, School of Health Sciences, Ahvaz Jundishapur University of Medical Sciences, Ahvaz, Iran; 6 Department of Psychology, Shahid Chamran University of Ahvaz, Ahvaz, Iran; 7 Student Research Committee, Ahvaz Jundishapur University of Medical Sciences, Ahvaz, Iran

**Keywords:** Sleep quality, DASH, Healthy Eating Index

## Abstract

**Background:**

Numerous factors such as nutrition and diet can affect the quality of sleep of people, especially employees. The aim of this study was to investigate the relationship between university employees' sleep quality and their dietary quality scores (HEI, DII and DASH score).

**Materials and Methods:**

This cross-sectional study was performed on 211 employees with a mean age of 38.75±11.31. Nutritional status of individuals was determined through the Food Frequency Questionnaire (FFQ) and to assess sleep quality, the Pittsburgh Sleep Quality Index (PSQI) was used. The quality of sleep decreases with increasing Pittsburgh index score. The calculated dietary quality scores include DASH Index, Healthy Nutrition Index (HEI) and Diet Inflammation Index (DII).

**Results:**

The results of this study after adjusting for confounding showed a significant positive relationship between DASH diet score and sleep duration (p <0.001). There was a significant negative relationship between HEI score and total score of PSQI (P = 0.003). Also, HEI score had a significant positive relationship with sleep duration in the unmodified and modified models (p <0.001), and a significant negative relationship was seen in unadjusted and modified model between DII score and sleep duration (p <0.001).

**Conclusion:**

It can be concluded that with increasing the diet quality in employees, the sleep quality also increases. This was the first study in south of Iran that examined the quality of sleep and diet of employees and the result of this study can affect the general health and improve the quality of foods consumed by employees.

## Introduction

Human health such as cardio-metabolic and mental health is related to sleeping habits, suggested by epidemiological evidence(1, 2). Most of evidence suggest that sleep duration and quality can influence on health of variety of systems in human body(3). Sleep is also very important for strengthening memory, improving vision, maintaining body temperature, maintaining and recovering energy (4), and restoration of the brain energy Metabolism (5). Therefore, sleep disorders may significantly affect the occupational, physical, cognitive and social performance of people and impair their quality of life (6).

Hormonal disruption, metabolic impairment and inflammatory process can play a role in the relationship between sleep and health status (7, 8). The quality of diet can affect sleep quality and health of individuals and for this reason, has recently been considered by researchers(9).

Studies have shown that diet plays an important role in causing or modulating inflammation in the body. Accordingly, it has been shown that following a Western diet increases inflammation, and following healthy diets such as the Mediterranean diet can be effective in reducing inflammation(10,11).

CRP, -glutamyl transferase (GGT), carotenoids, uric acid, vitamin C, and vitamin D, are several biomarkers of inflammation and antioxidants, that have been related to sleep quality parameters and duration(12). Evidence shows that there is an association between diet quality and inflammation, and a healthy dietary pattern can result in lower CRP levels(13).

Various diet quality scores have been developed to assess adherence to desirable priori-defined diets and patterns comprehensively and to investigate the health effects of these diets(14).

The Dietary Inflammatory Index (DII®) is a literature-derived score that has been developed to evaluate the inflammatory potential of the diet and link diet to inflammation. It takes into account six inflammatory markers (i.e., CRP, IL-1beta, IL-4, IL-6, IL-10, and TNF-alpha). The DII has proven to be of value for its association with health status in the general population(14, 15).

Of the other priori-defined dietary patterns, the Dietary Approaches to Stop Hypertension (DASH) diet, which recommends higher intakes of whole grains, fruits, vegetables, nuts, seeds, legumes, and low-fat dairy, and lower intakes of processed meat, sodium, and sweetened beverages, was originally developed to manage high blood pressure(16). The other score for evaluating the dietary quality is HEI score. The HEI is designed to examine the overall quality of diets, its adaptation and compatibility and coordination with dietary guidelines and food pyramid in 1995. This index is designed to evaluate diet quality in different societies with different dietary patterns(17).

Technical-administrative employees at universities mainly perform office tasks, in some cases involving much responsibility and demanding high levels of concentration. As a result, this population of workers might be more exposed to situations which might interfere with the duration of sleep. Since the quality of sleep of employees affects their performance and communication with clients, and given that the role of nutrition in employees' sleep quality has not been investigated to date, and also because of the important role of nutrition in health and prevention of chronic diseases, it was hypothesized that the quality of diet can affect the quality of their sleep. Therefore, this study was conducted to investigate the indices of dietary quality (Dietary Inflammatory Score, HEI score and DASH score) and their relationship with sleep quality of employees. The results derived from this study can significantly help to promote the health status of the employees. The aim of this study was to investigate the relationship between sleep quality and food quality indices on employees of Ahvaz Jundishapour University of Medical Sciences.

## Materials and Methods

Food Frequency questionnaire (FFQ) was used to assess their food intake and Pittsburgh questionnaire was used to assess sleep quality. Their initial information was also recorded and their height and weight were measured. Then, through GAM analysis, the relationship between sleep quality scores and nutritional indicators was determined. The detailed methods and of this study are given below.

**Participants**: Among 525 employees, 431 filled the consent form. Therefore, 275 of them had complete diet data, however after considering total energy intakes within the range of 800–4200 kcal, then 211 of participants was taken into the final analysis. Cluster random sampling from different departments in university. Participants' information was kept strictly confidential.

This descriptive-analytic study was conducted on 211 employees using cluster random sampling from different departments in Ahvaz Jundishapur University of Medical Sciences, aged between 18–50 who met the inclusion criteria. The inclusion criteria were willingness to participate in the study and age between 18–50 years. The exclusion criteria were reluctance to participate in the study, history of chronic disease, following vegan diet, pregnancy and lactation, taking certain medications.

The sample size was determined based on BMI (mean = 28.9 and SD = 4.5) as the primary outcome from a study conducted by Muscogiuri et al.(18);

The sample size was calculated using single population formula and assuming α = 0.05 and confidence level of 95% as 162 subjects. Considering the withdrawal rate of 35%, 218 subjects were recruited.

**Anthropometric and physical assessment**: Body weight and height was measured in the participants. Body weight was measured using a Seca scale, with an accuracy of 100 grams. Height was measured using a Seca stadiometer with an accuracy of 0.5cm. then BMI was calculated by dividing body weight by the height square.

**Dietary assessments**: The used food intake was obtained from the participants, by trained dietitians, through a structured interview. To determine typical food intakes, a valid and reliable 147- item semi quantitative FFQ with standard servings was used(19). the intake frequency of each food item was asked on a daily, weekly or monthly basis during the past year, and converted to the gram. Total energy and nutrient intake were then calculated using Nutritionist IV software as modified for Iranian foods.

**DII score**: For developing the latest DII, the relationship between food components or parameters and specific inflammatory markers was reviewed in the literature, published in 2010. Each article was assigned a positive or negative value, based on the effect of the food parameter on inflammatory markers (+1 = pro inflammatory relationship, 0= no significant relationship with inflammatory markers, -1 = anti-inflammatory relationship). Based on the number of pro-inflammatory and anti-inflammatory articles, an inflammatory effect score for each food parameter was calculated. First of all, each participant's dietary intake was linked to the global dataset, for calculating the DII score. Then the mean intake for each of the 45 food parameters was provided (12).

**HEI calculation**: By summing the sub scores in 13 categories, the total HEI score is calculated: (the score range in parentheses): total vegetables (0–5), greens and beans (0–5), total fruit (0–5), whole fruit (0–5), whole grains (0–10), total dairy (0–10), total protein (0–5), seafood and plant protein (0–5), fatty acids (0–10), sodium, refined grains (0–10), and “empty” calories from solid fats, alcohol, and added sugars (0–20). A better score, shows a better dietary quality (20).

**DASH score calculation**: DASH score, reward points for high intakes of five food groups, such as fruits, vegetables, nuts and seeds and legumes, low-fat dairy products and whole grains, according to quantiles ranking (i.e., participants in the lowest quintiles receive 1 point, those in the 2nd, 3rd, and 4th quintiles receive 2, 3, and 4 points respectively, and the highest quintiles, 5 points). Intakes of sodium, sweetened beverages, red meat and processed meat had the lowest score (i.e., the lowest quintiles are assigned 5 points and the highest quintiles, 1 point) (21).

**Sleep quality measurements**: To assess the sleep quality, The Pittsburg questionnaire (PSQI) was used. The Pittsburg questionnaire has 7 scales that include mental quality of sleep (ration of duration of useful sleep from time spent in bed), sleep disorders (waking up at night), dose of medicine measures sleep deprivation and dysfunction. The score of each scale is between 0–3 and the score of 3 in each scale determines the maximum negative. The overall score of this questionnaire is 0 to 21 and the overall score of 6 and above indicates the inadequacy of sleep quality.

**Statistical analysis**: Distributions of demographic, lifestyle behaviors, and dietary characteristics were compared across sleep quality score and time in bed (hr/d). Hence, we used chi-square test for categorical variables and one-way ANOVA test for continues variables.

The Generalized Additive Models (GAM) procedure was applied to the dataset of 211 without any missing data by smoothening the effect of covariates into crud and adjusted models. Moreover, the adjusted Model include age, sex and total daily energy intake.

GAM uses a link function to establish a relationship between the mean of the response variable and a smoothed function of the explanatory variable(s).

We used SPSS version16 and R version 3.6.2 to perform the analyses. P values <0.05 were considered significant. The FFQ analysis and nutrients were estimated using NUT IV software (Nutritionist IV).

**Ethical approval**: The study protocols were fully explained to the participants. The study protocols were approved by the ethics committee of AJUMS (IR.AJUMS.REC.1399.717). Each subject was given an informed consent to sign.

## Results

The results of the present study indicated that the demographic characteristics of the participants were not significantly associated with sum sleep and time in bed. The relationship between demographic characteristics and these variables are presented in Table 1 and Table 2. About 22.3% of males and 18.4% of females had sum-sleep less than 5 hours and 85% of males and 95% of females had time in bed less than 6 hours.

**Table 1:** Characteristics, diet quality scores and food intakes according to sum-sleep quality score

Variable	Sum sleep quality score		
	
	<5 sum score	≥5 sum score	P-value^1^
Weight (kg)	75(12.17)	74.91(13.34)	0.96
Age (years)	39.25(6.54)	39.94(7.52)	0.57
Hight(cm)	157.84(43.84)	174.46(125.72)	0.38
BMI (kg/m2)	26.08(3.81)	26.01(3.69)	0.97
**Sex (N) (%)**			0.47
Male	23(22.3)	80(77.7)	
female	21(18.4)	93(83.7)	
**Marital status (N) (%)**			0.004
Married	32(19)	136(81)	
Single	7(16.3)	36(83.7)	
Divorced	4(100)	0	
**Education status (N) (%)**			0.14
<12 years	1(6.3)	15(93.5)	
>12 years	43(21.7)	155(78.3)	
**Smoking status (N) (%)**			0.76
Yes	3(23.1)	10(74.9)	
No	40(19.7)	163(80.3)	
**Income status (N) (%)**			0.93
strongly satisfied	3(21.4)	11(78.6)	
partially satisfied	22(21.8)	79(78.2)	
strongly unsatisfied	14(20.3)	55(79.7)	
partially unsatisfied	4(16)	21(84)	
DII score	0.29(2.40)	-0.02(2.10)	0.41
DASH score	20.35(4.07)	20.65(3.92)	0.66
HEI score	62.83(7.16)	64.12(8.54)	0.37
Protein%	14.26(2.47)	13.94(2.07)	0.38
Carbohydrate%	62.09(7.23)	25.11(8.33)	0.68
Fat%	25.68(7.92)	25.11(8.33)	0.68
Energy (kcal/d)	2286.63(725.60)	2493.33(734.91)	0.06

DII= dietary inflammatory index, DASH= Dietary Approaches to Stop Hypertension, HEI=Healthy Eating Index

1 Based on chi-square test for categorical variables and one-way ANOVA test for continues variables.

Continuous variables are shown as means ± SDs, and categorical variables are shown as percentages. p < 0.05 was considered statistically significance

**Table 2:** . Characteristics, diet quality scores and food intakes according to Time in bed

Variable	Time in bed		P-value^1^
		
	<6 hour	≥6 hour	
Weight (kg)	74.82(13.18)	75.50(12.71)	0.77
Age (years)	39.99(7.20)	38.86(7.89)	0.39
Hight(cm)	172.79(124.22)	163.82(29.39)	0.66
BMI (kg/m2)	25.93(3.66)	26.46(3.97)	0.44
Sex (N) (%)			0.50
Male	85(82.5)	18(17.5)	
female	95(83.3)	19(16.7)	
Marital status (N) (%)			0.004
Married	146(86.9)	22(13.1)	
Single	31(72.1)	12(27.9)	
Divorced	2(50)	2(50)	
Education status (N) (%)			0.63
<12 years	14(87.5)	2(12.5)	
>12 years	164(82.8)	34(17.2)	
Smoking status (N) (%)			0.55
Yes	10(76.9)	3(23.1)	
No	169(83.3)	34(16.7)	
Income status (N) (%)			0.22
strongly satisfied	9 (64.3)	5 (35.7)	
partially satisfied	84 (83.2)	17 (16.8)	
strongly unsatisfied	60 (87)	9 (13)	
partially unsatisfied	20 (80)	5 (20)	
DII score	0.05(2.12)	-0.01(2.37)	0.86
DASH score	20.46(3.96)	21.21(3.87)	0.32
HEI score	63.65(8.11)	63.90(8.33)	0.87
Protein%	13.99(2.18)	14.08(2.04)	0.82
Carbohydrate%	62.92(8.26)	62.11(7.30)	0.58
Fat%	25.59(8.33)	25.91(7.83)	0.58
Energy (kcal/d)	2496.46(737.98)	2232.43(695.25)	0.04

DII= dietary inflammatory index, DASH= Dietary Approaches to Stop Hypertension, HEI=Healthy Eating Index

1 Based on chi-square test for categorical variables and one-way ANOVA test for continues variables.

Continuous variables are shown as means ± SDs, and categorical variables are shown as percentages. p < 0.05 was considered statistically significance.

In none adjusted model, there was not any significant relationship between any of the DASH quartiles and sum sleep. But, the p-value for second quartile was 0.06 that showed the relationship could be strong (Table 3).

**Table 3:** The Generalized Additive Models associations between baseline Healthy Eating Index score, sum sleep and Time in bed

	Model 0^a^				Model 1^b^			

Sum sleep	β	SE	t-value	P-value	β	SE	t-value	P-value
HEI Score	-0.11	0.001	-32.07	<0.001	-0.09	0.03	-2.98	**<0.05**
Q2 HEI Score	0.48	0.83	0.57	0.56	7.68	8.58	0.89	0.37
Q3 HEI Score	-0.11	1.21	-0.09	0.92	1.76	1.23	0.14	0.88
Q4 HEI Score	0.48	1.78	0.27	0.78	8.30	1.82	0.45	0.65
**Time in bed**								
HEI Score	0.08	0.001	50.55	<0.001	0.08	0.01	5.55	**<0.001**
Q2 HEI Score	-0.23	0.48	-0.47	0.63	-0.35	0.47	-0.75	0.45
Q3 HEI Score	0.06	0.75	0.08	0.93	-0.16	0.73	-0.22	0.82
Q4 HEI Score	0.37	1.00	0.37	0.70	0.11	0.98	0.11	0.90

a) Model 0, GAM generalized additive model without adjustment

b) Model I, GAM generalized additive model with adjustment for energy intake, age, BMI

HEI=Healthy Eating Index, Q= Quartile, SE=Standard error

Also, the results showed that there is a strong positive association between DASH score and time in bed, in adjusted and none adjusted model (P<0.001). The negative relationship between HEI and sum sleep and time in bed, in adjusted and non-adjusted model was strongly significant (P<0.001). There was not any significancy in the quartiles (Table 4). The relationship between DII score and time in bed and sum sleep is presented in Table 5. The results showed that there is a strong negative relation between DII and time in bed and sum sleep, in adjusted and non-adjusted model (P<0.001). The relationship between each quartile was examined, but no significance was observed.

**Table 4:** The Generalized Additive Models associations between baseline DASH score, sum sleep and Time in bed

	Model 0^a^				Model 1^b^			

Sum sleep	β	SE	t-value	P-value	β	SE	t-value	P-value
DASH Score	-0.05	0.06	-0.80	0.42	-5.59	7.03	-0.79	0.42
Q2 DASH Score	-1.82	1.15	-1.15	0.11	-1.37	7.27	-1.88	0.06
Q3 DASH Score	-2.37	1.68	-1.40	0.16	-7.16	8.57	-1.55	0.12
Q4 DASH Score	-2.11	2.29	-.91	0.36	-7.16	7.69	-0.93	0.35
**Time in bed**								
DASH Score	0.24	0.006	37.84	<0.001	2.05	5.703	3.61	**<0.001**
Q2 DASH Score	0.58	0.52	1.12	0.26	0.70	0.57	1.35	0.17
Q3 DASH Score	0.23	0.75	0.30	0.76	0.15	0.75	0.21	0.83
Q4 DASH Score	0.03	1.03	0.02	0.97	0.13	1.03	0.12	0.89

a Model 0, GAM generalized additive model without adjustment

b Model I, GAM generalized additive model with adjustment for energy intake, age, BMI

DASH= Dietary Approaches to Stop Hypertension, Q= Quartile, SE=Standard error

**Table 5:** The Generalized Additive Models associations between baseline Dietary Inflammatory Index score, sum sleep and Time in bed

	Model 0^a^				Model 1^b^			

Sum sleep	β	SE	t-value	P-value	β	SE	t-value	P-value
DII Score	-0.10	0.12	-0.79	0.42	-1.02	1.30	-0.78	0.43
Q2 DII Score	0.02	1.37	0.01	0.98	5.04	8.22	0.61	0.54
Q3 DII Score	0.10	2.15	0.05	0.96	-1.393	8.02	-0.24	0.80
Q4 DII Score	0.82	2.79	0.29	0.76	-4.90	8.03	-0.61	0.54
**Time in bed**								
DII Score	-0.13	0.02	-5.46	<0.001	-1.35	0.23	-4.21	**<0.001**
Q2 DII Score	0.09	0.64	0.15	0.87	1.14	6.68	0.17	0.86
Q3 DII Score	-0.36	1.01	-0.35	0.72	-5.26	1.04	-0.50	0.61
Q4 DII Score	-0.63	1.29	-0.48	0.62	-7.43	1.31	-0.56	0.57

a Model 0, GAM generalized additive model without adjustment

b Model I, GAM generalized additive model with adjustment for energy intake, age, BMI

HEI= Dietary Inflammatory Index, Q=Quartile, SE=Standard error

## Discussion

In the present study, the relationship between DASH, DII and HEI score with sum sleep and time in bed was assessed, in the employees. The results of the present study showed that there is a positive association between DASH score and time in 21bed; also, there is a strong negative association between HEI score and sum sleep, and a strong positive association between HEI score and time in bed. But we noticed a positive association between DII score and sum sleep. It means that by increasing HEI and DASH score, sleep quality increases, but by increasing DII score, the sleep quality decreases. There are several studies that have surveyed the relationship between sleep quality and diet quality.

Similar to our study, Liang et al. In a study of 3941 American adults found that following a dash diet could effectively improve sleep quality and sleep duration(22). Also, similar to our study, Mossavar-Rahmani in a study of 2,140 Spanish adults found that increasing the Healthy Eating Index effectively increased sleep duration and sleep efficiency(23). Also, another study conducted on 1500 adults in Spain, showed a lower variation in sleep duration, in the individuals, adherent to the Mediterranean diet(24) and the results of this study were in line with the present study. Another study conducted by Jaussent et.al found a positive effect of Mediterranean pattern on specific aspects of sleeping(25). Consumption of fruits, legumes, nuts and fish is an important factor in the indication of Healthy Eating Index and following a DASH diet(21). Various studies have shown that these factors can be effective in improving sleep quality. Jyväkorpi et al. Showed that eating vegetables helps to sleep better(26). Hakkarainen et al. Also showed that people with insomnia eat less vegetables(27). Kurotani et al. And Cao Y et al. Showed that consumption of vegetables, fruits and legumes is associated with better sleep quality(28). Liang et al. Also found that reduced consumption of vegetables, nuts, and legumes was associated with reduced sleep duration(22). Other studies also confirm that consumption of fruits and vegetables is directly related to sleep duration and sleep quality(24).

In addition, del Brutto et al., Showed that fish consumption can be effective in improving sleep duration(29). Kurotani et al., Also showed that fish consumption can reduce the PQS (poor quality of sleep) score(28) and nutrition showed that fish is related to sleep timing(30). And all the factors mentioned above, are parts of HEI and DASH diet, and the present study expressed that higher HEI and DASH scores improved sleep quality.

Refined grains, Sodium, Added Sugars and Saturated Fats are factors that reduction of their consumption can increase the score of the Healthy Eating Index(21). Sodium is also important in determining the score of dash diet(31). Mossavar-Rahmani et al., showed that among the components of AHEI-2010, decreased sodium intake was most strongly associated with higher sleep efficiency (23). Grandne et al., indicated that sugar consumption was directly related to poor sleep quality(32). Jyväkorpi et al., Also showed that consumption of Sugars and Saturated Fats are associated to poor sleep quality(26). Moreover, other animal and small human experimental studies have shown that high-saturated-fat diets may influence in circadian rhythms(33).

In a recent study by Lopes et al, it was shown that the DII score was only positively associated with daytime sleepiness as a component of PSQI(34). In the study by Godos et al., participants in the highest quartile of DII score were more likely to have poor sleep(35).

An interventional study also showed that an anti-inflammatory diet including increased intake of fruits, vegetables, lean protein, and reduced intake of added sugars and SFAs could improve sleep quality(36).

There are some possible explanations for the mechanisms underlying the inverse association between sleep quality and the healthy pattern, one of the explanations is that proteins include tryptophan that is a component of serotonin production and neurosecretory hormone melatonin. sleep cycle, either directly or indirectly, and brain function is controlled by Serotonin. Generally, serotonin promotes wakefulness, also regulates sleep through changes in the concentration of melatonin. Melatonin exerts a hypnotic effect through thermoregulatory mechanisms by lowering the core body temperature, reducing arousal and increasing sleep-propensity(37).

Also, vitamin B6, pyridoxine, is required for the synthesis of serotonin from tryptophan. The 5-Hydroxytryptophan is an intermediate in this process, and is converted to serotonin by a pyridoxal 5′-dependent enzyme. In relation, dietary niacin is involved in leaving more tryptophan to be used for the synthesis of serotonin.

In addition, folate is involved in the metabolism of monoamines like serotonin in the brain. The N-3 fatty acids are required to convert serotonin to melatonin by the enzyme arylalkylamine-Nacetyl transferase (38).

Other nutrients such as Magnesium enhances the secretion of melatonin from the pineal gland by stimulating serotonin N-acetyltransferase activity, which is the key enzyme in melatonin synthesis(39). In addition, oxidative stress can lead to insomnia. Diets with rich in antioxidant vitamins including vitamin C, have a high score of the healthy dietary pattern, which may decrease the levels of oxidative stress and prevent the development of DIS. Therefore, the above-mentioned nutrients or their combination might have a beneficial role in initiating sleep.

Increased HEI and DASH scores are associated with increased protein intake resulting in increased tryptophan. Diets with higher DASH and HEI score, contain more dark green vegetables, fruits, nuts and seeds(20, 21).

As a result, it increases the intake of magnesium, folate and vitamin C and other antioxidants from the diet, which are important factors in regulating effective hormones in sleep(28). Diets with higher HEI and DASH scores have more variety of foods, and contain whole grains that are high in B vitamins, so they can help individuals with their sleep quality.

Increasing fish intake increases omega-3 fatty acids and B12 intake, which improve sleep quality. B12 increases melatonin receptors in the brain. Fish also contains glycine, which can improve sleep satisfaction.

Decreasing the DII score of a diet indicates the anti-inflammatory properties of the diet, which together with increasing the consumption of nutrients such as magnesium, niacin B6, B12, folate, vitamin C and omega 3, are effective in regulating melatonin(37).

Other anti-inflammatory agents that have been shown to be effective in improving quality and sleep time include potassium, fiber, and calcium(22, 28).

Studies have also shown that some nutrients that have inflammatory properties in the DII index, play an important role in reducing sleep duration and sleep quality. These nutrients include total fat, SFA and cholesterol(40).

One of the reasons that increased DII scores, results in decreased sleep quality, may be because of the increase in CRP levels, that is related to increased sleep apnea. Also, researchers have found that inflammation and inflammatory factors are positively linked to oxidative stress; these factors include advanced-glycation end products like erythrulose that decreases sleep quality.

One of the strengths of this study is that no such study has been conducted in the south of Iran, about dietary scores and sleep quality, also no study has been done to evaluate the food and nutrition of employees and that the results of this study provide conditions for other studies. But there are limitations to this study, too. We could not cover different ethnicities and our sample size was limited to Jundishapur University and other university centers in the province were not surveyed; also, the sample size of the study reduced the original sample size by excluding very high and low calories.

The results from this study showed that there is a significant relationship between dietary health scores, such as HEI and DASH score and sleep quality derived from PQSI in staff of AJUMS. Also, the results showed that increasing DII score, which is caused by increasing the consumption of inflammatory foods, reduces the quality of sleep. This study was the first study that investigated the relationship between dietary quality indices and sleep quality in Ahvaz. The results can help the employees to change their diet and lifestyle and can avoid inflammatory diets to improve their sleep for more accurate performance. We suggest other researchers to conduct other studies with a larger sample size and different population so that the results can be assigned to other populations.
